# Mean echogenicity and area of puborectalis muscle in women with stress urinary incontinence during pregnancy and after delivery

**DOI:** 10.1007/s00192-016-3030-8

**Published:** 2016-05-05

**Authors:** Maria K. van de Waarsenburg, Mariëlla I. J. Withagen, Anique T. M. Grob, Karlijn J. Schweitzer, Greetje A. van Veelen, Carl H. van der Vaart

**Affiliations:** 1 Department of Obstetrics and Gynecology, University Medical Centre Utrecht, Heidelberglaan 100 Huispostnummer F05.126, 3584 CX Utrecht, The Netherlands; 2 MIRA Institute for Biomedical Technology and Technical Medicine, University of Twente, Enschede, Netherlands

**Keywords:** 3D/4D, Echogenicity, Muscle area, Perineal ultrasound, Stress urinary incontinence

## Abstract

**Introduction and hypothesis:**

Pregnancy and childbirth are risk factors for the development of stress urinary incontinence (SUI). Urinary continence depends on normal urethral support, which is provided by normal levator ani muscle function. Our objective was to compare mean echogenicity and the area of the puborectalis muscle between women with and those without SUI during and after their first pregnancy.

**Methods:**

We examined 280 nulliparous women at a gestational age of 12 weeks, 36 weeks, and 6 months after delivery. They filled out the validated Urogenital Distress Inventory and underwent perineal ultrasounds. SUI was considered present if the woman answered positively to the question “do you experience urine leakage related to physical activity, coughing, or sneezing?” Mean echogenicity of the puborectalis muscle (MEP) and puborectalis muscle area (PMA) were calculated. The MEP and PMA during pregnancy and after delivery in women with and without SUI were compared using independent Student’s* t* test.

**Results:**

After delivery the MEP was higher in women with SUI if the pelvic floor was at rest or in contraction, with effect sizes of 0.30 and 0.31 respectively. No difference was found in the area of the puborectalis muscle between women with and those without SUI.

**Conclusions:**

Women with SUI after delivery had a statistically significant higher mean echogenicity of the puborectalis muscle compared with non-SUI women when the pelvic floor was at rest and in contraction; the effect sizes were small. This higher MEP is indicative of a relatively higher intramuscular extracellular matrix component and could represent diminished contractile function.

## Introduction

Pregnancy and childbirth are risk factors for the development of stress urinary incontinence (SUI) [1, 2]. Urinary continence depends on normal urethral support, which is provided by normal levator ani muscle function, more specifically the puborectalis muscle part, and an intact endopelvic fascia. Damage to this urethral support during vaginal delivery can result in loss of function and increased mobility of the bladder neck [3–5], which is the main contributing factor to SUI [6].

During pregnancy SUI has been associated with the width of the hiatal area, and after delivery with the positioning of the bladder neck [5]. The observation that during pregnancy a large hiatal area is associated with SUI raises the question whether this is related to structural abnormalities of the puborectalis muscle, which forms the boundaries of the genital hiatus. Normal functioning of a muscle is, among other things, dependent on its volume and structural integrity. Regarding true volume measurements of the puborectalis muscle we would need adequate 3-D delineation, which is not readily available. In 2-D planes the area of the puborectalis muscle can be assessed and, within its limitations, be indicative of the volume. With regard to the structure of muscles we know that atrophic muscles and muscles with increasing extracellular matrix (ECM) content (collagen) have poor contractility force [7]. This structural composition of a muscle can be indirectly assessed with ultrasound, especially by measuring echogenicity [8]. Echogenicity of the muscle represents the ratio between muscle cells and ECM, and was recently shown to change during pregnancy and after delivery (unpublished data).

In this study, we set out to assess the association between the puborectalis muscle area (PMA) and SUI symptoms and that between the mean echogenicity of the puborectalis muscle (MEP) and SUI symptoms during and after first pregnancy.

## Materials and methods

This study is a secondary analysis of a prospective observational study on the association between pelvic floor symptoms and changes in pelvic floor anatomy during and after first pregnancy [5]. Two hundred eighty nulliparous women with a singleton pregnancy and good knowledge of the Dutch language were included in the original study. Exclusion criteria were a medical history of urinary or fecal incontinence, prolapse or anti‐incontinence surgery, connective tissue diseases, neurological disorders and an inability to perform a maximum Valsalva maneuver because of cardiac or pulmonary disease. The Institutional Human Research Ethics Committee approved the study (reference 08/299) and all women gave informed consent.

The participants were invited for 3D/4D transperineal ultrasound examination at a gestational age of 12 weeks and 36 weeks and 6 months after delivery. The examinations were performed by two observers, one of the observers had 6 years’ experience with 3D/4D transperineal ultrasound and the other observer was trained by the experienced observer. We have previously published data on their intra- and interobserver reliability [9]. A GE Voluson 730 Expert ultrasound system (GE Healthcare, Hoevelaken, the Netherlands) with an RAB 4‐8 MHz curved array 3D/4D ultrasound transducer was used. It was crucial that the intensity values were kept constant, as described by Scholten et al. [10]. We used gain 15, power 100, Harmonics mid, contrast 8, grey map 4, persistence 8, and enhance 3. The women had an empty bladder. Volume imaging datasets were obtained at rest, on maximum pelvic floor muscle contraction and on maximum Valsalva maneuver.

After storage on a hard disk, offline analysis was performed using the 4D View 7.0 (GE Medical Systems Kretztechnik, Zipf, Austria) and Matlab® R2010a (MathWorks, Natick, MA, USA) software. The plane of minimal hiatal dimensions in axial position was selected and exported as previously described by Dietz et al. [11]. A semi-automated method was used to delineate the puborectalis muscle and measure PMA and MEP. This method had been tested previously and proved to be reliable [12].

Pelvic floor symptoms and physical complaints were scored at every ultrasound examination. SUI was present when a woman answered positively to the Urogenital Distress Inventory question “do you experience urine leakage related to physical activity, coughing, or sneezing” [13, 14].

The association between the MEP and body mass index (before pregnancy), the mode of delivery (vaginal vs caesarean section), the duration of the second stage of labor (<60 min and ≥60 min), the use of oxytocin (yes/no) during delivery, the mean birth weight, and the use of pain relief (drugs or epidural) were assessed for potential confounding effects.

Statistical analysis was performed using SPSS version 20.0 for Windows. The MEP and PMA during pregnancy and after delivery between women with and without SUI were compared using independent Student’s* t* test. Statistical significance was based on two-sided tests, with *p* < 0.05 considered significant. To determine the magnitude of the effect we calculated the effect size of the statistically significant findings using Cohen’s d.

## Results

Of the 280 women, 26 cases were excluded, leaving 254 women to be studied. Excluded were women who had been included incorrectly because of a twin pregnancy (*n* = 1) and a neurological disorder (*n* = 1). Other reasons for exclusion were immature labor at 19.9 weeks’ gestation (*n* = 1), loss to follow-up, and/or at least one out of three ultrasound volume datasets (rest, contraction or Valsalva) missing (*n* = 17), and the symphyses was located outside the view of the ultrasound images (*n* = 6).

**Fig. 1:**
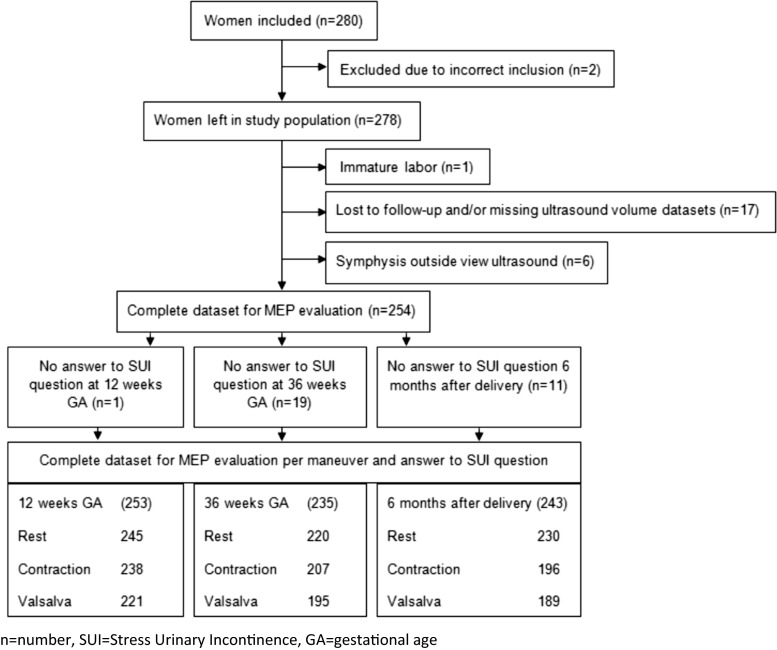
Flow chart

The flowchart (Fig. 1) shows the distribution of women with complete datasets who could be analyzed for each timeframe. Patient characteristics are described in Table 1.

**Table 1:** Patient characteristics

Variables	*N* = 254
Age at first ultrasound, years; mean (SD)	31.1 (4.1)
BMI at first ultrasound, kg/m^2^; median (range)	22.4 (17.9–40.4)
Gestational age at first ultrasound, weeks; median (range)	13.0 (8.4–21.0)
Time period after delivery at third visit, weeks; median (range)	27.0 (23.6–57.4)
Gestational age at delivery, weeks; median (range)	40.2 (33.0–42.6)
Delivery mode;* n* (%)	249
Spontaneous vaginal	157 (63.1)
Operative vaginal	45 (18.1)
Elective CS	11 (4.4)
Emergency CS	36 (14.4)
Birth weight, g; median (range)	3,365 (1,590–4,750)

*CS* cesarean section

The relationship between MEP and SUI is shown in Table 2. During pregnancy no statistically significant differences in the mean echogenicity of the women with SUI and those without SUI at the different maneuvers were found. Six months after delivery there was a statistically significantly higher MEP in women with SUI compared with women without SUI when the pelvic floor was at rest (*p* = 0.03) and when the pelvic floor was in contraction (*p* = 0.04), with effect sizes of 0.30 and 0.31 respectively.

**Table 2:** Mean echogenicity of the puborectalis muscle (*MEP*) in relation to stress urinary incontinence (*SUI*) during pregnancy and after delivery

Gestational age and maneuver (*n*)	Women with SUI		Women without SUI		*p* value
	*n* (%)	MEP mean ± SD	*n* (%)	MEP mean ± SD	
12 weeks (253)	44 (17.4)		209 (82.6)		
Rest (245)	44	141.0 ± 20.6	201	141.0 ± 20.1	0.99
Contraction (238)	43	136.3 ± 22.3	195	132.2 ± 20.6	0.25
Valsalva (221)	38	134.7 ± 21.9	183	134.7 ± 21.2	0.99
36 weeks (235)	112 (47.7)		123 (52.3)		
Rest (220)	104	147.0 ± 19.8	116	149.2 ± 19.8	0.42
Contraction (207)	97	138.1 ± 19.9	110	139.0 ± 21.9	0.76
Valsalva (195)	89	133.6 ± 24.6	106	135.1 ± 21.7	0.65
6 months after delivery (244)	90 (36.9)		154 (63.1)		
Rest (230)	87	132.1 ± 20.9	143	126.2 ±19.8	0.03
Contraction (196)	72	125.6 ± 23.7	124	118.6 ± 22.6	0.04
Valsalva (189)	71	118.7 ± 22.0	118	115.0 ± 21.3	0.25

The relationship between PMA and SUI is shown in Table 3. No significant differences were found in the PMA during pregnancy and after delivery in women with and those without SUI.

**Table 3:** Puborectalis muscle area (*PMA*) in relation to SUI during pregnancy and after delivery

Gestational age and maneuver (n)	Women with SUI		Women without SUI		*p* value
	*n* (%)	PMA (cm^2^) mean ± SD	*n* (%)	PMA (cm^2^) mean ± SD	
12 weeks (253)	44 (17.4)		209 (82.6)		
Rest (245)	44	5.9 ± 1.4	201	5.6 ± 1.3	0.12
Contraction (235)	42	5.5 ± 1.2	193	5.0 ± 1.2	0.11
Valsalva (222)	38	6.0 ± 1.5	184	5.8 ± 1.3	0.32
36 weeks (235)	112 (47.7)		123 (52.3)		
Rest (219)	104	5.9 ± 1.4	115	5.8 ± 1.2	0.82
Contraction (207)	97	5.4 ± 1.2	110	5.2 ± 1.3	0.27
Valsalva (194)	89	6.6 ± 1.5	105	6.2 ± 1.3	0.10
6 months after delivery (244)	90 (36.9)		154 (63.1)		
Rest (230)	87	5.6 ± 1.2	143	5.4 ± 1.5	0. 43
Contraction (192)	69	5.2 ± 1.2	123	4.8 ± 1.4	0.10
Valsalva (191)	72	5.9 ± 1.4	119	5.9 ± 1.5	0.86

None of the potential confounding factors was significantly associated with the MEP.

## Discussion

We set out to assess the association between MEP/PMA and SUI during and after first pregnancy. We found that the MEP in women with SUI after delivery was statistically significantly higher than that in women without SUI. However, effect sizes were low, indicating that the clinical relevance is questionable and that MEP cannot be used to differentiate women with SUI from those without.

A possible limitation of our study is the absence of pre-pregnancy clinical and ultrasound data. We were only able to look at associations between SUI and MEP and PMA at different time points during and after pregnancy. Changes in MEP and PMA that occurred between pre-pregnant and early pregnant status may have provided extra information on the association between these parameters and SUI. We know from epidemiological studies that childbirth is the major risk factor for developing stress urinary incontinence symptoms. Therefore, we feel it is not an obvious limitation to look at the association between stress urinary incontinence symptoms and ultrasound findings postpartum without having knowledge of pre-pregnancy data. Another limitation is the fact that we had to use the PMA as a surrogate marker for puborectalis muscle volume.

The presence of levator avulsions could be a cause of a smaller PMA, as the avulsion area, which is darker, would not have been incorporated into our semiautomatic muscle outline method. However, we previously demonstrated that the reliability of detecting levator avulsions in this particular population of postpartum women, when assessed in a muliticenter, multiobserver setting, is poor [15]. This showed us that assessing levator avulsions in the population under study cannot be reliably done and therefore we considered it inappropriate to use it as a potential confounding factor in our present paper.

We used a symptom-based assessment of SUI according to the ICS standardization, in line with a previous study [16, 17]. We did not consider it appropriate to perform a standardized stress test in pregnant women to confirm incontinence as a sign or to perform multichannel urodynamics to confirm incontinence as a condition. Our results have to be viewed from this symptom-based SUI perspective.

The strengths of this study are the prospective design and the use of identical ultrasound settings during the examinations, which made echogenicity analyses possible.

The higher MEP, i.e., brighter muscle on ultrasound images, is indicative of a change in muscle tissue composition. The ratio between muscle cells and ECM expresses itself in the echogenicity (grey-scale) values on ultrasound [8]. Muscle cells appear dark on ultrasound, whereas the extracellular matrix (ECM), containing mainly collagen and fat, appears bright. An increase in echogenicity has been associated with disease progression in children with neuromuscular disease [8], and was shown to be associated with a decrease in muscle strength [18]. The echogenicity of a muscle increases with ageing and after major or recurrent minor injuries [19–21]. Muscle injuries can lead to scar formation and loss of contractility function [20, 21]. Our finding that the MEP after delivery in women with SUI is higher than in non-SUI women indicates that the muscle composition has changed in favor of the ECM. This may affect the contractile force of the puborectalis muscle and diminish urethral support, which may present as hypermobility of the urethra and consequently SUI. We did not observe differences in MEP between women with and those without SUI during pregnancy. The obvious reason is that delivery injury has not yet occurred and no scar tissue, which affects echogenicity, has been formed.

The PMA was not related to SUI, whereas the hiatal area in a previous analysis of our data was [5]. The hypothesis was that a larger hiatal area is associated with a smaller sized puborectalis muscle. In that respect we would expect that SUI also was associated with a smaller sized puborectalis muscle. Our observation that there is no association between SUI and PMA is limited by the fact that we did not measure the true volume of the puborectalis muscle, which would have needed accurate 3-D volume information. Since muscle volume is associated with muscle strength [22], the absence of an association between the PMA and SUI has to be interpreted with caution.

We previously demonstrated that SUI was associated with a larger hiatal area during pregnancy, and with a more dorsal and caudal positioning of the bladder neck after childbirth [5]. We suggested that the pathophysiology of SUI was different during pregnancy compared with after delivery. This hypothesis is challenged by other groups, who demonstrated different associations from ours between SUI and the hiatal area or bladder neck positioning both during and after pregnancy [23, 24]. However, our current findings on the MEP support the view that the mechanism behind SUI is different during pregnancy from that after delivery.

Although statistically significant, the difference between the MEP in women with SUI and those without was small. This may be related to the moment of scanning, which was 6 months after delivery. We do not know how many women were breastfeeding or had returned to their normal menstrual cycle by that time point [25, 26]. Estrogens play an important role in the wound healing process and low estrogen status, as in ageing, is associated with poorer healing [27]. Therefore, depending on their estrogen status, women may have been in different stages of recovery from their delivery trauma during the time of scanning. In fully developed scar tissue the echogenicity of the scar tissue does not change between rest and contraction [20]. However, our data show that there is a difference in MEP between rest and contraction in SUI women. This may indicate that the trauma to the puborectalis muscle was mild with little scar tissue formation [28], or that the complete scar tissue formation in major trauma has not yet fully occurred. We could not demonstrate a significant association between the MEP at Valsalva after delivery between women with and those without SUI. This may be caused by the fact that the Valsalva maneuver, or pushing, is a passive stretching of the muscle. In contrast, little information is available on what happens with muscle echogenicity during contraction in Valsalva. One of the reasons may be that performing a true Valsalva maneuver is much more difficult than performing a contraction. There are large individual differences that affect the data obtained on MEP and PMA. In addition, Mulder and coworkers showed that performing a maximal Valsalva maneuver requires a long, forceful bearing down [29]. During pregnancy and after delivery in particular, women may be reluctant to do so.

In conclusion, women with SUI after delivery were shown to have a statistically significantly higher MEP than non-SUI women when the pelvic floor was at rest and in contraction, although the effect sizes were small. This higher MEP is indicative of a relatively higher intramuscular ECM component and could represent diminished contractile function.
